# Spatial multi-omics analyses of the tumor immune microenvironment

**DOI:** 10.1186/s12929-022-00879-y

**Published:** 2022-11-14

**Authors:** Wan-Chen Hsieh, Bugi Ratno Budiarto, Yi-Fu Wang, Chih-Yu Lin, Mao-Chun Gwo, Dorothy Kazuno So, Yi-Shiuan Tzeng, Shih-Yu Chen

**Affiliations:** 1 Institute of Biomedical Sciences, Academia Sinica, Taipei, Taiwan; 2 Genome and Systems Biology Degree Program, Academia Sinica and National Taiwan University, Taipei, Taiwan; 3 Institute of Biotechnology, College of Bio-Resources and Agriculture, National Taiwan University, Taipei, Taiwan; 4 Taiwan International Graduate Program in Molecular Medicine, National Yang Ming Chiao Tung University and Academia Sinica, Taipei, Taiwan

**Keywords:** Spatial, Multi-omics, Tumor-immune microenvironment (TIME), Heterogeneity

## Abstract

In the past decade, single-cell technologies have revealed the heterogeneity of the tumor-immune microenvironment at the genomic, transcriptomic, and proteomic levels and have furthered our understanding of the mechanisms of tumor development. Single-cell technologies have also been used to identify potential biomarkers. However, spatial information about the tumor-immune microenvironment such as cell locations and cell–cell interactomes is lost in these approaches. Recently, spatial multi-omics technologies have been used to study transcriptomes, proteomes, and metabolomes of tumor-immune microenvironments in several types of cancer, and the data obtained from these methods has been combined with immunohistochemistry and multiparameter analysis to yield markers of cancer progression. Here, we review numerous cutting-edge spatial ‘omics techniques, their application to study of the tumor-immune microenvironment, and remaining technical challenges.

## Background

### The tumor-immune microenvironment and the therapeutic challenge

The inter-related, co-existing, and competitive nature of interactions between tumor cells, the surrounding tissue, and infiltrating innate and adaptive immune cells results in a unique environment that varies by tumor type and is highly adapted to the tumor behavior. This complex ecosystem is composed of tumor cells, immune cells, stromal cells, fibroblasts, extracellular matrix, and blood vessels, and is referred to as the tumor-immune microenvironment (TIME). In the TIME, dynamic and bidirectional interactions occur between cells of various types through communication signals such as secreted molecules, proteins, and vesicles [1]. Tumor cells, as well as immune and stromal cells, utilize specific metabolic pathways to survive in the oxygen- and nutrient-limiting environment of a tumor [2].

Tumor-infiltrating T cells, which recognize tumor-specific antigens and kill tumor cells, are modulated by multiple signals emitted by tumor cells and by myeloid cells [3]. Monocytes and macrophages, specifically tumor-associated macrophages, can suppress or modulate the tumor killing by T cells [4]. Other non-immune cells, such as endothelial cells and fibroblasts, are critical regulators of tumor progression in the TIME [5, 6]. Endothelial cells build blood vessels necessary for transfer of nutrients to the tumor. Cancer-associated fibroblasts not only lay down the matrix but also actively participate in immune modulation.

Recently developed therapeutic strategies that leverage the immune system to inhibit tumor growth have proven effective in some solid tumors. Although immune checkpoint blockers (ICBs) such as anti-CTLA-4 antibody ipilimumab, anti-PD-1 antibodies nivolumab and pembrolizumab, and anti-PD-L1 antibody atezolizumab have been evaluated in the treatment of many different cancers [7], ICB therapy works only on certain types of cancers. Loss of tumor-expressing MHC class I molecules, which initiate the T cell response, insufficient numbers of T cells in the tumor mass, and the presence of immunosuppressive cells or factors can all limit efficacy of ICBs. Thus, the TIME strongly influences the effectiveness of ICB therapies [8]. To understand how to harness the immune system to inhibit tumor progression, a detailed understanding of immune cell distribution and function is imperative.

### Single-cell technologies reveal the heterogeneity of the tumor-immune microenvironment

The development of single-cell detection platforms has enabled deep profiling of the heterogeneity of tumors and the immune system both within individual tumors and between patients [9, 10]. Genomic single-cell sequencing methods, which detect mutations at the DNA level, have been used to confirmed co-existence of dangerous mutations in individual cells, a resolution that cannot be achieved by bulk sequencing [11, 12]. Single-cell RNA sequencing (scRNA-seq) enables profiling of transcriptomes in cells of the TIME [13]. These technologies have yielded high-resolution and unbiased profiling of cancerous cells, T cells, myeloid cells, and stromal cells and have revealed a vast heterogeneity of immune profiles across tumor types [14, 15].

Single-cell proteome detection platforms with multiplex capacity have also been developed. For example, mass cytometry (also known as cytometry by time-of-flight or CyTOF) methods can detect over 40 cellular markers at one time [16, 17]. CD4^+^ and CD8^+^ T cells, tumor-associated macrophages, and cells that express immune checkpoint markers, the hierarchy of hematopoietic stem cell differentiation, functional heterogeneity and signaling in T and natural killer (NK) cells have been revealed using the CyTOF platform [18–24].

Experiments using single-cell technologies have convincingly demonstrated that tumor masses usually contain multiple genetically defined subclones of cells with distinct sets of gene mutations and different transcript profiles. Immune cells, with vastly different transcript profiles characteristic of progenitor, active, exhausted, and suppressed cells, coexist with tumor. These data have raised critical questions: How do these immune players with vastly different functions coexist in the same environment? Are these cells equally distributed in the tumor mass, or do they segregate into clusters with distinct spatial and biological features? To answer these questions, a single-cell dataset embedding histological structure information is needed.

### A new era of single-cell-level histological research

**Fig. 1:**
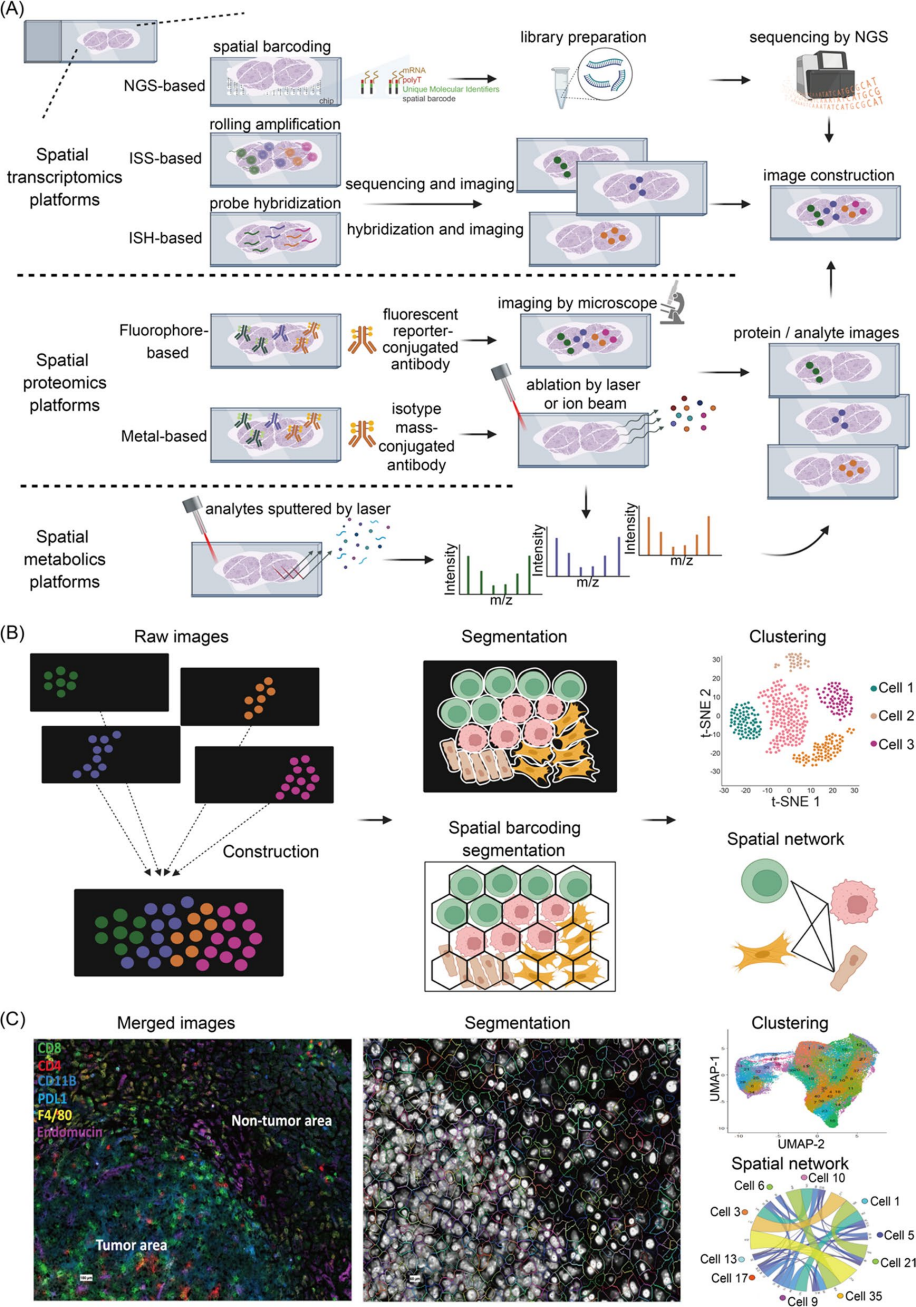
Schematics of the spatial multi-omics technologies, analysis workflow, and example data. **A** Schematics of workflows for spatial transcriptomics, proteomics, and metabolic analyses. Upper: Spatial transcriptomics platforms are classified into those based on next-generation sequencing and those based on in situ hybridization and in situ sequencing. The next-generation sequencing-based approaches use chips covered by a matrix of the barcoded oligonucleotides to capture mRNAs from the overlaid tissue. After tissue removal and probe harvest, a cDNA library with coordinate barcodes is prepared and sequenced. The in situ hybridization-based methods use fluorescently labeled probes that hybridize to the target transcripts. The in situ sequencing method uses probes to capture target transcripts, and sequencing is performed after rolling circle amplification. Middle: The spatial proteomic platforms utilize fluorescent reporters or metal-conjugated antibodies to recognize target proteins. For fluorescent reporters, repeated imaging and stripping to remove probes allow detection of many antibodies. In the methods that employ metal-conjugated antibodies, tissue is systematically ablated by a laser or an ion beam and analysis by mass spectrometry yields spatial and molecular information. Lower: For the spatial metabolomics, metabolites can be ionized and detected after sputtering from a spot or pixel on the tissue by a laser. **B** An example of a data analysis workflow for image processing and downstream analyses (clustering, spatial network analysis, or evaluation of cell–cell interactions) applicable to spatial multi-omics data. During image processing, information on the position of each cell is obtained by algorithmic definition. Clustering and neighborhood analyses can then be performed on the segmented images to obtain information about how cell types interact. **C** Example of results obtained from a multiplexed immunofluorescence imaging study performed using the CODEX method. The composite image of six key antibodies staining from the panel is shown on the left. The cell segmentation is shown in the middle. Cellular clustering and neighborhood analyses were performed, as shown on the right. Clusters are color coded. A chord diagram is used to represent the interactions between cell clusters. The size of the arc is proportional to the strength of the cell–cell interactions

Long before the development of the spatial ‘omics, it was known that certain histological patterns within tumors are highly linked to patient prognosis. For example, peritumoral T cell and B cell infiltration in colorectal cancer patients is correlated with positive prognosis, whereas the depletion of lymphocytes in the tumor core is an indicator of poor prognosis [25]. Further, stromal infiltration of T cells is linked to good prognosis for specific types of breast cancer [26, 27]. To better understand spatial correlates with prognosis researchers have used multiplexed immunohistochemistry (IHC), immunofluorescence (IF) and laser capture microdissection-based IHC/IF with auxiliary tools to select tumor regions for analyses of expression patterns of protein and RNA transcripts [28]. These types of histological analyses are limited by resolution and problems related to bias in sample selection. Recent technological advancements in solid-phase sequencing and multiplex imaging have enabled multiplexed detection of transcripts, proteins, and metabolites in high-resolution images (Fig. 1). In the following sections, we will review recent developments in spatial multi-omics, research applications in tumor tissue exploration, and current challenges.

### Spatial transcriptomics methods

The most commonly used methods for spatially resolved transcriptomic analyses are listed in Table 1, and a schematic of the spatial transcriptomics workflow is shown in Fig. 1A (upper panel). Depending on the technique, these experiments use either fresh-frozen (FF) or formalin-fixed, paraffin-embedded (FFPE) tissues. The main strategies for spatial transcriptomics are based on next-generation sequencing (NGS) and on fluorescence in situ hybridization (FISH) [29–31]. With NGS techniques, transcripts are encoded with position information prior to sequencing. For example, with the 10 × Genomics Visium platform, chips containing spatially barcoded oligo(dT) are used to capture mRNA from the tissue overlaid on the chip and then processed for sequencing yielding unbiased spatial transcriptomic data [32]. However, due to the low numbers of transcripts captured, transcript abundances from multiple neighboring cells are aggregated for downstream analyses, so the effective spatial resolution is not at the single-cell level.

**Table 1:** Spatial transcriptomic platforms

Spatial transcriptomic technique	Biomolecule target	Read-out	Resolution	Coverage	Number of targets	Tissue preparation	References
NGS based							
10X Visium	RNA	Sequencing	55 μm	Full	> 10,000	FFPE, FF	[32]
Slide-seq	RNA	Sequencing	10 μm	Full	> 10,000	FF	[33]
Slide-seq V2	RNA	Sequencing	10 μm	Full	> 10,000	FF	[34]
XYZseq	RNA	Sequencing	Single cell in 500 μm microwells	Full	10,000	FFPE, FF	[35]
HDST	RNA	Sequencing	2 μm	Full	> 10,000	FF	[36]
Stereo-seq	RNA	Sequencing	0.22 μm on chip	Full	> 10,000	FF	[38]
ZipSeq	RNA	Sequencing	Single cell	Full	> 10,000	live cells	[40]
Pick-seq	RNA	Sequencing	5–20 cells	Full	> 10,000	FFPE, FF	[44]
DBiT-seq	RNA	Sequencing	10 μm	Full	> 10,000	FF	[41]
Seq-Scope	RNA	Sequencing	~ 0.6 μm on chip	Full	> 10,000	FF	[37]
In situ sequencing based							
FISSEQ	RNA	Sequencing by ligation	Sub-cellular	Full	16,000	FFPE, FF	[46]
STARmap	RNA	Sequencing by ligation	Single cell	Targeted	1020	FF	[47]
In situ hybridization based							
MERFISH	RNA	Cyclic imaging	Sub-cellular	Targeted	> 10,000	FF	[48]
Seq-FISH +	RNA	Cyclic imaging	Sub-cellular	Targeted	> 10,000	FF	[52]
DSP	RNA	Sequencing	200 μm	Targeted	1600	FFPE	[43]

Other spatial transcriptomic techniques rely on spatially barcoded probes conjugated to beads to capture RNA from the tissue samples. For example, Slide-seq is a method for transferring RNA from tissue sections onto a surface covered in DNA-barcoded beads with known positions, allowing the locations of each RNA transcript to be inferred by sequencing [33]. Slide-seq V2 has higher RNA capture efficiency than Slide-seq; the efficiency is about 50% that of scRNA-seq [34]. XYZseq encodes spatial information by physically partitioning tissue sections into an array of microwells containing barcoded primers for reverse transcription primers [35]. The High-Definition Spatial Transcriptomics (HDST) method uses microwell-based fluorescence spatial indexing beads to capture transcripts, and fluorescence signals reveal transcript positions in tissue samples. The barcoded RNA is sequenced, and sequencing data is mapped to fluorescence data. Cyclic sequencing of individual transcripts can be used to increase resolution. For both Slide-seq and HDST, the size of beads and the capture efficiency of RNA transcripts determines the resolution of spatial information: The diameter of beads used in HDST is around 2 μm, and the beads used for Slide-seq are 10 μm in diameter. HDST captures more targets and provides higher resolution than Slide-seq [36].

Recently, spatial transcriptomic approaches with higher transcriptome capture efficiencies have been developed to enable capture of sub-µm-resolution images. In Seq-Scope, tissue is attached to an RNA-capturing array with a dense arrangement of barcoded clusters [37]. mRNAs captured from the tissue are used as the template to generate cDNAs for NGS analysis. In Stereo-seq, circular amplified DNA nanoballs containing barcode sequences are generated and dispersed onto the etched chips with patterned array [38]. An mRNA capture sequence is linked to the spatial barcode and used to capture the mRNA released from the tissue section overlaid on the chip. Each dot is 220 nm in diameter, and there is 500 or 715 nm between the dots; however, the lateral diffusion of mRNA during the capture step is more than 5 μm. Optimized Stereo-seq can read-out 1450 unique molecular identifiers per 10 μm diameter (a bin of 14 × 14 DNA nanoballs) providing spatial transcriptomic data for a tissue area as large as several cm^2^. Although detection of low-abundance copy transcripts is not currently possible, this technique has been applied to analyze mouse embryonic development and the transcriptomes of solid tumors at near single-cell resolution [38, 39].

Spatially encoded barcodes can also be allocated to individual cells through light-based printing of DNA barcodes onto the surface of cells. In ZipSeq, patterned illumination and photocaged oligonucleotides are used to serially print barcodes (the so-called zipcodes) onto live cells within tissues [40]. This annealed zipcode terminates in a polyA sequence that streamlines subsequent cDNA library construction. For sequencing, the tissue sample is dissociated into single cells and scRNA-seq is performed. The final analysis combines mapping transcripts onto zipcoded regions.

In Deterministic Barcoding in Tissue sequencing (DBiT-seq), two perpendicular microfluidic chips with parallel channels are sequentially placed against the tissue section to introduce oligo-dT-tagged with combinations of spatial barcodes for each tissue pixel [41]. The tissue is then digested to recover spatially barcoded cDNAs, and libraries are prepared and sequenced. Spatial transcriptome mapping of the developing eye in an E10 mouse embryo was achieved using DBiT-seq with 10-μm resolution. By combining DBiT-seq with immunofluorescence staining or scRNA-seq, a better understanding of specific cell types could be achieved.

Methods to profile the transcriptome within a region of interest (ROIs) have also been developed. With the Digital Spatial Profiler (DSP), the abundances of proteins or RNAs within an ROI are measured by counting unique barcoded oligonucleotides assigned to each target of interest. Antibodies or mRNA hybridization probes are conjugated with barcoded photocleavage oligonucleotides and used to stain the tissues. Next, photocleavage is induced by UV light, and the released oligonucleotides, which encode spatial information, are collected for NGS analysis [42]. An optimized DSP method was recently used to detect 44 proteins and 96 transcripts simultaneously [43]. Similarly, Pick-seq can be used to sequence RNAs in an ROI of a tissue sample [44]. The ROI, selected based on immunofluorescence, is isolated by aspiration into a liquid-filled 40-μm bore needle. Subsequently, cells are lysed, and cDNA is prepared and sequenced.

Imaging-based strategies, which combine interactive in situ hybridization or in situ sequencing with high-resolution microscopy, can achieve subcellular spatial resolution and can potentially provide genome-wide transcriptomic information. However, these methods are usually technically demanding, require iterative workflows, sophisticated image analysis processes, and large collections of probes. In methods based on in situ sequencing, probes are used to hybridize pre-selected RNA targets to allow reverse transcription. Rolling circle amplification, sequencing-by-ligation, sequencing-by synthesis, or sequencing by hybridization can be used as read-out [45]. Fluorescent in situ sequencing (FISSEQ) is an untargeted gene profiling method in which transcripts are reverse transcribed, and rolling circle amplification is used to generate cDNA amplicon nanoballs of 200–400 nm in diameter within the cell. The amplicons are then sequenced using Supported Oligonucleotide Ligation and Detection (SOLiD) technology to yield in situ transcriptomic data [46]. Targeted in situ transcriptomics images have been obtained using Spatially resolved Transcript Amplicon Readout mapping (STARmap), which is a combination of hydrogel tissue chemistry methods with targeted signal amplification- And a targeted method, Sequencing with Error-reduction by Dynamic Annealing and Ligation (SEDAL) method, was used to simultaneously map 1020 genes in mouse V1 neocortex [47].

The method known as MERFISH, for multiplexed error-robust FISH [48], is an advanced method derived from single-molecule FISH (smFISH) [49, 50]. Multiplexing results from rounds of hybridization, imaging, and stripping. In smFISH due to numerous single image merges, the error of each image also accumulates. The scheme used in MERFISH allows detection of errors and replacement with valid sequence [48, 51]. MERFISH has been used to spatially resolve 10,050 genes simultaneously at subcellular resolution and to identify specific gene subsets that are enriched in subcellular compartments. seqFISH + applies a complex in situ hybridization technique to obtain higher dimensional results than MERFISH [52]. The primary oligonucleotide probes have sequence complementary to RNAs of interest and a readout region for secondary probe binding. The secondary probes are conjugated to combinatorial fluorophore labels used in imaging to potentially allow detection of 24,000 genes in single cells.

### Spatial proteomics methods

The idea that arose from traditional IHC that multiple proteins could be detected in a single step staining procedure prompted development of highly multiplexed spatial proteomic detection (Table 2 and Fig. 1A, middle). Most proteomics platforms either employ fluorophores or metal tags. The method are similar in terms of spatial resolution, cell throughput, number of the molecular targets, and the temporal dynamics. For fluorescence-based methods, a procedure called iterative image acquisition is applied to the FF or FFPE tissue of interest through cyclic addition and removal of fluorescently labeled primary antibodies. Examples include MACSima Imaging Cyclic Staining (MICS) [53], tissue-based cyclic immunofluorescence (t-CycIF) [54, 55], Co-Detection by IndEXing (CODEX) [56], Signal Amplification By Exchange Reaction (Immuno-SABER) [57], and InSituPlex [58]. The choice of fluorophores or design of oligonucleotides is important for the success of spatial detection using these iterative methods. The antibodies are conjugated to fluorophores that have minimal spectral overlap (maximal Stokes shift), to indexing oligonucleotides (CODEX), to orthogonal DNA concatemers (Immuno-SABER), or amplified oligonucleotides (InSituPlex) to provide specificity and sensitivity during the iterative detection process. To bleach or inactivate the label, t-CycIF and MICS use gentle conditions such as photobleaching with specialized antibodies (REAfinity or REAdye_lease) or a specialized removal reagent (REAlease). In Multi Omic Single-scan Assay with Integrated Combinatorial Analysis (MOSAICA), secondary probes are conjugated to the combinatorial fluorophore labels used in imaging [59]. Spatial results are captured by fluorescence lifetime imaging and microscopy (FLIM). Using FLIM, fluorescence spectral detection and lifetime measurements are obtained for each pixel. Fluorescence spectral and lifetime data are processed by a machine learning-based decoding method, and phasor analysis is utilized for mapping spectral and temporal information to original images yielding three-dimensional tissue images. CODEX has the advantage of not requiring an amplification enzyme or specialized buffers, which makes it less costly and less time-consuming [56].

**Table 2:** Spatial proteomics platforms

Spatial proteomic technique	Biomolecule target	Read-out	Resolution	Number of targets	Tissue preparation	References
Fluorophore-based						
MICS	Protein	Cyclic imaging	Sub-cellular	100	FFPE	[53]
t-CycIF	Protein	Cyclic imaging	Single cell	60	FFPE	[54, 55]
CODEX	Protein	Cyclic imaging	Single cell	60	FFPE, FF	[56]
Immuno-SABER	Protein	Cyclic imaging	Sub-cellular	10	FFPE, FF	[57]
InSituPlex	Protein	Cyclic imaging	Sub-cellular	5	FFPE	[58]
MOSAICA	Protein/ Nucleic acid	Spectral and time resolved fluorescence imaging	Single cell	Up to 10	FFPE	[59]
Metal-based						
IMC	Protein	Mass cytometry	1 μm	40	FFPE, FF	[60]
MIBI	Protein	Mass cytometry	260 nm	40	FFPE, FF	[61]

The metal tag-based spatial proteomics methods are imaging mass cytometry (IMC) [60] and Multiplex Ion Beam Imaging (MIBI) [61]. In these strategies, metal-conjugated antibodies are used to stain tissue samples, and target proteins are detected using mass spectrometry based on the abundances of isotopic reporter masses released from the tissue upon ablation with a laser beam or ion beams. The output is as a non-overlapping mass signal integration for each measured cell. A single laser is used in IMC to ablate tissue. The two source ion beams employed in MIBI result in higher spatial resolution than is obtained with IMC [61–63]. Both IMC and MIBI have been used to study tumor samples from pre-clinical and clinical studies and have significantly contributed to our understanding of the highly complex architecture of TIME at a cellular level and its role in tumorigenesis [62, 64–70]. Multiplexed fluorescence- and metal-based tagging methods can be employed to detect protein and mRNA within the same tissue. Co-detection strategies aim to reveal both genotypic and phenotypic information about a cell simultaneously [71].

### Spatial metabolic methods

Mass spectrometry is a robust technique for multiplexed analysis of proteins, natural products, and metabolic derivatives [72, 73]. Since classical mass spectroscopy methods do not provide spatial information, mass spectrometry-based imaging strategies have been developed that employ different ionization methods [74]. These include matrix-assisted laser desorption/ionization mass spectrometry (MALDI) [75, 76], desorption electrospray ionization (DESI) [77, 78], and secondary ion mass spectrometry (SIMS) [79, 80]. These platforms allow label-free detection, quantification, and mapping of multiple metabolites including small molecules, lipids, peptides, organic compounds, and elemental ions in cells and tissues (Table 3, Fig. 1A, lower panel).

**Table 3:** Mass spectroscopy-based spatial metabolomic platforms

Spatial proteomic technique	Biomolecule target	Read-out	Resolution	Coverage of mass range	Tissue preparation	References
DESI	Metabolite	DESI	50–200 µm	0–2000 Da	Solid, frozen liquid	[78]
AFADESI	Metabolite	DESI	300–500 µm	0–2000 Da	Solid, frozen liquid	[89]
nanoDESI	Metabolite	DESI	10–15 µm	0–2000 Da	Solid, frozen liquid	[90]
MALDI	Metabolite	MALDI	2–10 µm	0–20,000 Da	Dried sample in matrix	[75, 76]
t-MALDI-2	Metabolite	MALDI	0.6–2 µm	0–20,000 Da	Dried sample in matrix	[84]
SIMS	Metabolite	SIMS	50 nm	0–1000 Da	Dried sample	[79, 80]
TOF–SIMS	Metabolite	SIMS	0–1 µm	0–10,000 Da	Dried sample	[80]
3D OrbiSIMS	Metabolite	SIMS	0.3 µm	0–1000 Da	Dried sample	[93]
SEAM	Metabolite	SIMS	1.5 µm	0–2000 Da	Cryosections	[94]

MALDI requires applying and crystallizing the sample onto a matrix. The resolution of current commercial MALDI instrumentation is about 10 μm, and atmospheric pressure MALDI provides resolution below 2 μm [81]. To improve ionization efficiency, MALDI-2 combines a laser post-ionization with MALDI [82]. Transmission-mode MALDI-2 (t-MALDI-2) provides resolution of 1–2 μm for detection of phospholipids and some biomolecules [83], and 0.6-μm spatial resolution was obtained by adapting the strategy to the Orbitrap mass analyzer [84].

DESI has been used to analyze drug, biological and metabolic molecules under ambient conditions [85–88]. For DESI, a matrix is not necessary; charged droplets and ions of solvent are sprayed directly onto the surface of analyte. The analyte on the surface is taken up by a stream of charged solvent droplets to form the multiply charged ions analyzed by mass spectroscopy. The spatial resolution of DESI is about 50–200 µm. Signal levels decrease and sensitivity is reduced when large areas of tissue are analyzed, such as whole-body sections of mice. To address this and expand the coverage of metabolites, air flow-assisted ionization was incorporated into the DESI workflow in a pipeline called air flow-assisted DESI (AFADESI) [89]. The resolution of AFADESI is 300–500 µm. Another DESI-based protocol, nanoDESI, which incorporates a solvent bridge between primary and nanospray capillaries, has spatial resolution of 10–15 µm [90].

SIMS facilitates soft ionization of analytes via a primary ion beam. As SIMS is a high vacuum technique, sample preparation for SIMS usually requires chemical or cryogenic fixation to maintain tissue integrity [91]. Several methods have been developed based on SIMS. TOF–SIMS combines the time-of-flight (TOF) and SIMS to obtain information on the molecular layers of a solid surface to increase resolution [80]. The spatial resolution of TOF–SIMS can reach 1 μm. 3D OrbiSIMS is label-free and has subcellular lateral resolution (0.3 μm) and a high mass resolving power [92, 93]. Despite limitations in terms of sample type and complicated sample preparation, 3D OrbiSIMS enables visualization of exogenous and endogenous metabolites in tissues in three-dimensions. The spatial single nuclear metabolomics (SEAM) method was developed to solve problems in segmentation and representation in SIMS data [94]. SEAM preserves the native state of samples with fast and minimal sample processing, providing in situ metabolic fingerprints and single nuclei clustering.

### Studies of tumor progression using spatial ‘omics

**Fig. 2:**
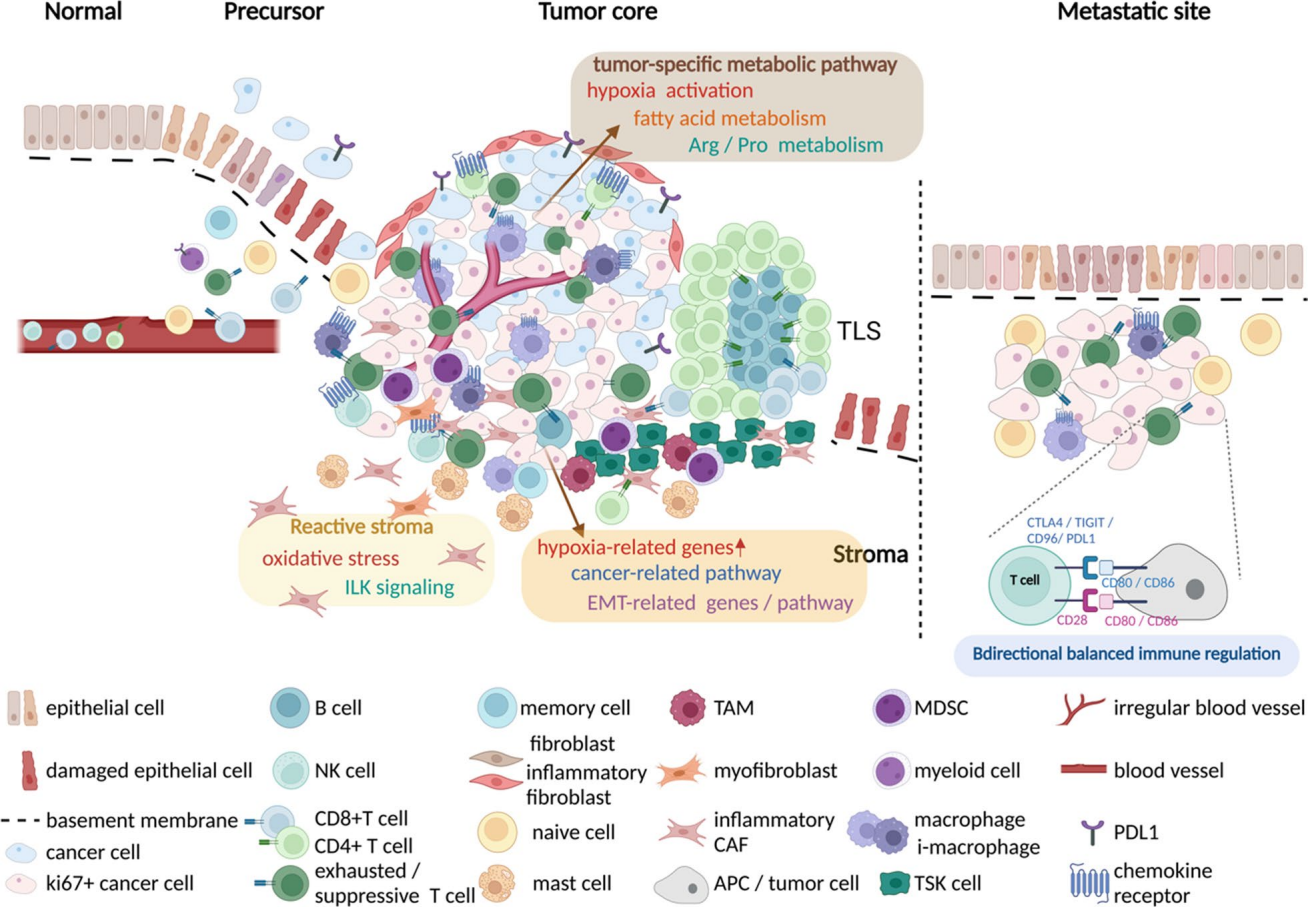
The TIME landscape revealed by spatial multi-omics. *EMT* epithelial-mesenchymal transition, *TAM* tumor-associated macrophage, *CAF* cancer-associated fibroblast, *APC* antigen-presenting cell, *MDSC* myeloid derived suppressor cell, i-macrophage, inhibitory macrophage, *TSK* tumor-specific keratinocyte

Researchers have used spatial ‘omics to zoom in on cancer, uncovering various features of the TIME. Studies of the genomes, transcriptomes, proteomes, and metabolomes of normal and cancerous tissues have revealed differences in cellular compositions in different compartments, tumor-immune cell interactomes, and correlates of tumor progression (Fig. 2). The following sections describe how spatial ‘omics tools have been used to image normal and tumor tissues in preclinical and clinical samples.

### Spatial imaging reveals compartmentalization of tumor and immune cells

Solid tumors are composed of tumor cells, immune cells, stromal cells, and a vascular system. These components are not evenly distributed. Tumor cell-rich and stroma-rich areas are evident under traditional histological examination. For example, breast cancer has well characterized histopathological features and serves a good model for spatial cellular profiling. This cancer has been studied using spatial transcriptomics [95–97] and high-dimensional antibody-based tissue profiling [67, 70]. Using scRNA-seq, Wu et al. profiled breast cancer tissue cell types and the spatial localization of tumor-associated immune and stromal cells [95]. Based on the spatial annotation, the authors found that T cells were mostly located in an area rich in lymphocytes and in an area composed of stromal cells and lymphocytes. The locations of *CD4*^+^ and *CD8*^+^ T cells were positively correlated with *APOE*^+^ macrophages which also express *PD-L1* and *PD-L2*, suggesting an active immunoregulation. At the invasive tumor front, both *cxcl10*-expressing macrophages and *CD8*^+^ T cells were detected.

In another study, Andersson et al. used spatial transcriptomics to profile HER2^+^ breast cancer tissue [96]. They discovered that tumor and immune cells are compartmentalized. Immune cells are either intermingled with connective tissue or clustered as a lymphoid organ structure, some surrounding the ductal carcinoma in situ (DCIS) area. Similar findings were reported by Salmen et al., who also used a spatial transcriptomics approach to study HER2^+^ breast cancer specimens [97]. They also found enrichment of T helper 2 and class-switched memory B cells in the DCIS area. In agreement with findings reported by Wu et al. [95], Salmen et al. detected strong associations between macrophages that express *CXCL9* and *CXCL10* and T cells and NK cells that express *CXCR3*.

Antibody-based, single-cell protein expression profiling using MIBI was used by Keren et al. to analyze locations of 36 proteins in specimens from patients with triple-negative breast cancer (TNBC) [70]. The proteins included those expressed by tumor cells, immune cells, and stromal cells. Functional markers for proliferation and epigenetic and immune regulation were also included. In agreement with spatial transcriptomics approaches, B cell presence was highly correlated with CD4^+^ and CD8^+^ T cells. A cell neighborhood analysis revealed a clear tumor-to tumor neighbor and immune-to-immune neighbor patterns. These findings reflect the tumor tissue compartmentalization seen in spatial transcriptomics data.

Spatial proteomics studies have also revealed this compartmentalization of tumor and immune cells in several other cancers. Topological distribution of immune cells within the hepatocellular carcinoma (HCC) specimens was studied by Sheng et al. using IMC [98]. Based on 36 antibodies identifying tumor, stromal, immune, and endothelial cells, they discovered several tumor-stromal-immune cell clusters that reflected coordinated cellular neighborhoods and compartmentalization of tumor and immune cells. Most immune cells (infiltrated macrophages, T and B cells) were absent from the cancerous region or limited to the perivascular region. Instead, these cells clustered within an immune-fibroblastic area. Sheng et al. also discovered a spatial relationship between macrophages and T cells. The liver-resident macrophages, the Kupffer cells, were enriched in the peritumoral area and were mainly immunosuppressive. T cells surrounding the Kupffer cells expressed higher level of PD-1 than those surrounding the infiltrating macrophages.

Chan et al. studied small cell lung cancer (SCLC) samples using the MIBI platform [99]. SCLC has long been considered an “immune-cold” cancer due to little infiltration of leukocytes into the tumor. Quantitatively, the immune-mixing score determined using MIBI was indeed lower within the SCLC tumors than in other types of cancer. The SCLC tumors expressed *NEUROD*, which was associated with poor prognosis, and Chan et al. also found that CD8^+^ T cells from SCLC tumor samples were more exhausted and had lower effector-like gene expression than did tumors of other subtypes.

Immunological features are used to classify patients with colorectal cancer (CRC) into either the Crohn’s-like reaction (CLR) subtype or the diffuse inflammatory infiltration (DII) subtype. The immune cell topologies in CRC samples of both subtypes were studied by Schurch et al. using CODEX [100]. Tertiary lymphoid structures (TLSs) are present at the tumor invasive front in CLR tumors but are absent in tumors from DII patients; the former patients have much longer overall survival. Cell neighborhood coordination was evaluated by analysis of the CODEX metadata. As suggested by histological analyses, CODEX analysis showed that immune-tumor cellular neighborhoods were more prevalent in the DII type than in the more compartmentalized CLR type. The CODEX images also showed that CD8^+^ T cell proliferation was more frequently observed in the CLR type tumors, whereas immunosuppressive regulatory T cell proliferation was more frequently found in the DII type. In CLR, direct communication between T cells and macrophages that express activity markers such as ICOS were frequently found in tumor boundary areas but not the bulk tumor areas. In contrast, immunosuppressive granulocytes were more active in the DII type than the CLR type tumor boundary areas. Schurch et al. also found higher frequencies of T cells in the bulk CLR-type tumors than in DII bulk tumors and enrichment of immunosuppressive granulocytes and macrophages in the DII subtype tumors. These data suggest that increased number of immunosuppressive cells and lack of compartmentalized interactions between immune subsets might lead to worse outcomes in DII patients compared to patients with CLR-type tumors.

### Immune cells show more exhaustion markers when distributed in the tumor parenchyma

**Fig. 3:**
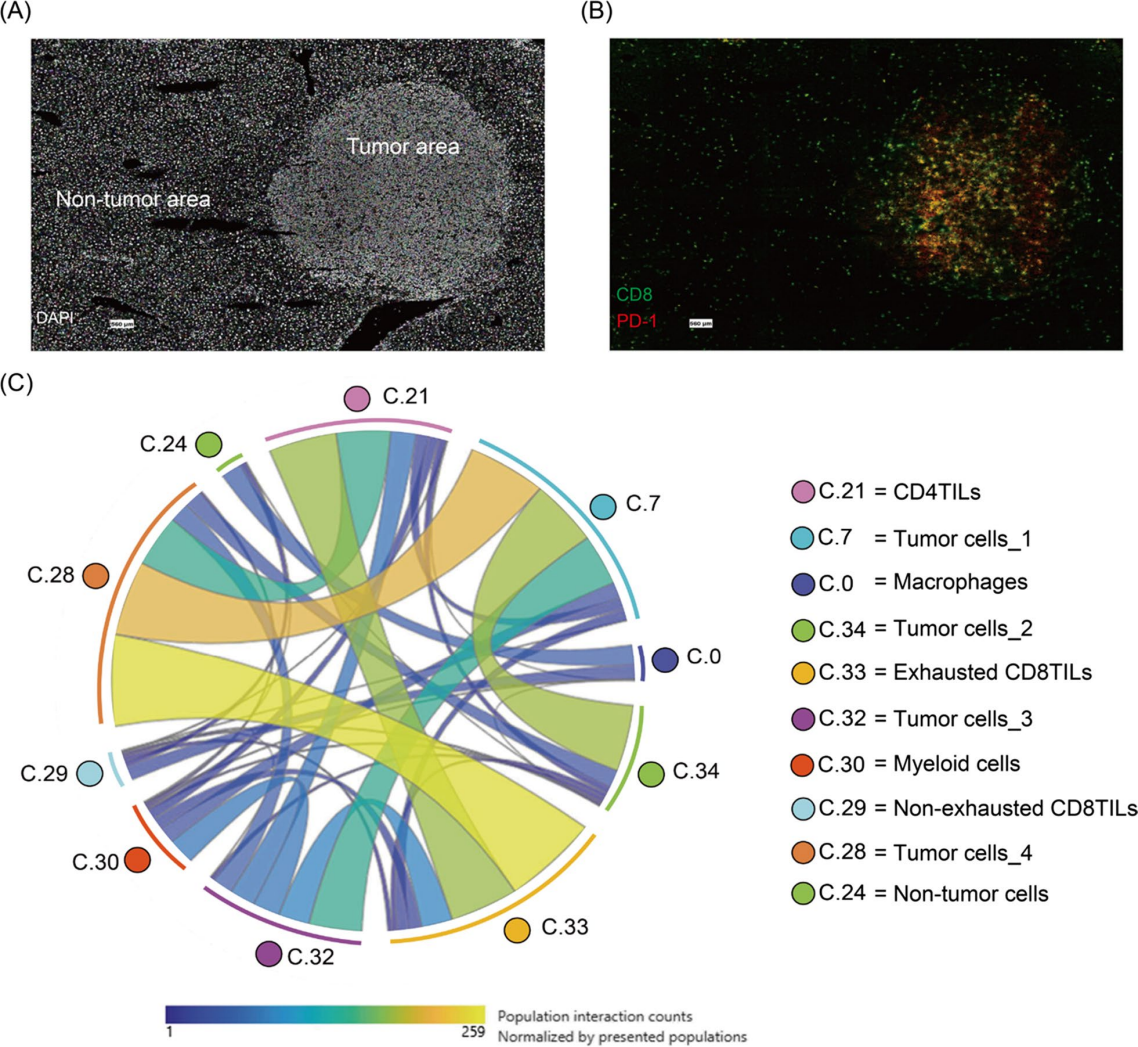
CODEX analysis of a mouse B cell lymphoma model reveals immune exhaustion in tumor core. **A** DAPI staining from the CODEX image stack, showing a clear tumor compartment. **B** Composite image of two markers extracted from the same CODEX image stack showing that the exhausted marker, PD-1 (red), co-localizes with CD8^+^ TILs (green) residing within the tumor area. **C** Cell–cell interactions based on CODEX neighborhood analysis; strong to weak correlations are light green to dark blue

In a spatially resolved transcriptomic analysis of tumor from patients with cholangiocarcinoma, Wu et al. observed that T cells in the tumor core expressed higher levels of exhaustion markers than those in the tumor boundary or peritumoral normal tissue [39]. The same pattern was detected in our recent unpublished CODEX analysis of a mouse model of B cell lymphoma (Fig. 3). We detected enrichment of exhausted CD8^+^ tumor-infiltrating lymphocytes (TILs) in the tumor compartment relative to the surrounding tissue (Fig. 3A and B). A cell neighborhood coordination analysis also showed that the cluster of exhausted CD8^+^ TILs (cluster 33) strongly interacts with two tumor cell clusters (clusters 28 and 32) as well as a cluster of CD4^+^ TILs (cluster 21) (Fig. 3C). In contrast, our data indicate that cells of the non-exhausted CD8^+^ TIL cluster (cluster 29) interact with macrophages (cluster 0) and non-tumor cells (cluster 24) (Fig. 3C).

### Spatial multi-omics approaches reveal active tumor-immune interactions at the invasive front

Invasive fronts, where tumor cells border immune cells, are usually the areas of active immune reactions. A spatial transcriptomics stratification reported by Anderssen et al. revealed more interaction among various cell types at the front than in the tumor core in HER2^+^ breast cancer [96]. Further, Anderssen et al. found that tumors in some patients are enriched for natural killer T cells, CD8^+^ effector memory T cells, T helper 1 cells, CD4^+^ naïve T cells, and memory B cells at the invasive front and that these patients have better survival outcomes than those without prominent infiltration. A spatial proteomics analysis of TNBC by Keren et al. revealed a gradient of histone methylation activity along the tumor-immune border, indicating an active chromatin status when tumor cells are close to the border [70]. They also detected CD11c^+^/CD11b^+^ myeloid-derived suppressor cells expressing PD-1, PD-L1, and IDO at the invasive front, suggesting that the front is an immunosuppressive site in this type of breast cancer. In agreement with Anderssen et al., Keren et al. showed that immune cell infiltration at the tumor-immune border was correlated with better prognosis.

In a study of HCC and intrahepatic cholangiocarcinoma (ICC) tumors, Wu et al. carefully examined the cellular composition of the tumor border using Stereo-seq [39]. They found that NK cells, T cells, and macrophages accumulate close to the boundary between the tumor and normal tissue. NK cells and T cells were detected within 250–500 μm from the border and express immune checkpoint genes. Macrophages, mainly anti-inflammatory (M2-like), were located within 250 μm of the border on the tumor side. These results indicate that there is a unique microenvironment at the invasive tumor front. Detailed subgrouping of the hepatocytes at the borders of these tumors revealed a group of hepatocytes that express cellular damaging markers including *SAA1* accumulate in the border region. Spatially, *SAA1*-expressing hepatocytes were detected along the border in close vicinity to macrophages. Since the recruited macrophages were mostly the M2-like phenotype, this recruitment might facilitate invasion of cancerous cells into normal tissue and, in the long run, worsen patient prognosis.

### Behaviors of the tumor-associated stromal cells and fibroblasts are spatially correlated

Cancer-associated stromal and fibroblastic cell compositions are heterogenous and have correlated spatial distributions in the tumor tissue. Combining scRNA-seq and spatial transcriptomics, Moncada et al. studied the cellular distribution and functional status of these cell types in pancreatic ductal adenocarcinoma tissue sections [101]. They identified a cancer cell cluster highly associated with fibroblasts in which both cancer cells and fibroblasts are enriched for inflammatory gene signatures. The spatial transcriptomics data also revealed that M1 inflammatory macrophages are associated with a cancer cell subpopulation that is enriched for stress-response genes and associated with inflammatory fibroblasts. Similarly in ICC tumors, fibroblasts that express matrix-forming genes and inflammatory, antigen-presenting features were present in the peritumoral area [98].

In breast cancer, cancer-associated fibroblasts (CAFs) have been mapped using a spatial transcriptomics approach and scRNA-seq [95]. The so-called inflammatory  CAFs (iCAFs) that express *ALDH1A1*, *KLF4*, *LEPR*, and *CXCL12* were distributed across the areas that contain both tumor and immune-stromal cells, whereas the myofibroblast CAFs were enriched only in the tumor cell-enriched area. The iCAFs were also anatomically correlated with areas enriched in memory and naïve B cells and *CD4*^+^ and *CD8*^+^ T cells. CAFs have long been considered immune regulatory cells that mobilize against tumor antigens. Analyses of spatial transcriptomic data revealed a strong chemokine, complement pathway and TGFβ-mediated cross-talk between the iCAFs and the neighboring T cells.

Risom et al. utilized the MIBI to profile the stromal component evolution from the early DCIS to invasive breast cancer [67]. They found that DCIS without progression was characterized by fewer fibroblasts and discontinuous myoepithelium surrounding the DCIS. In samples of tumors that progressed into invasive cancer, the DCIS was associated with active fibroblasts, fiber formation, and absence of myoepithelium. Further, E-Cadherin expression by the myoepithelium and its continuity around the tumor were highly associated with tumor malignancy.

### Spatial cellular profiling identifies tumor-associated tertiary lymphoid structures

TLSs are locations where tumor antigens are presented to T cells [102]. Due to the heterogeneous composition of the lymphoid structures, the identification of the tumor-associated TLSs can be difficult without the power of multiplexed immunostaining and transcriptomics information. TLSs harbor multiple types of immune cells with distinct functions. Spatial analyses can identify aggregates of B cells, T cells, and dendritic cells that are characteristic of these structures. Studies of breast tumors revealed a B cell-enriched TLS in TNBC patients and a TLS in HER^+^ breast cancer patients with a high degree of co-localization between B and T cells [70, 96]. A TLS structure was also found in the CLR type of CRC that is not present in the DII type; in the latter the PD-1^+^/ICOS^+^/CD4^+^ T cells were enriched in TLSs [100]. The presence of TLSs is an indicator of better prognosis for patients with CRC.

### Tumor tissue topological profiling identifies novel tumor biomarkers

Like other multi-omics approaches, spatial transcriptomics and scRNA-seq methods have the potential to lead to discovery of new tumor biomarkers. As an example of this potential, Gouin et al. used scRNA-seq, spatial transcriptomics methods, and CODEX to study specimens collected from 25 muscle-invasive bladder cancer patients [102]. From the single-cell clustering data, they discovered a novel biomarker, N-Cadherin 2 (encoded by *CDH12*), which is indicative of a cancerous epithelium cluster associated with stem-cell-like feature and poor prognosis. Cellular neighborhood analyses from spatial transcriptomics and CODEX both revealed that the T cells in the *CDH12*-expressing cancer cell vicinity expressed more exhaustion markers. These findings, supported by data from other types of cancer [99], show that tumor cells with more de-differentiated, more proliferative, and more aggressive features are usually associated in situ with exhausted T cells.

### The immune landscape of the metastatic tumor

The spatial interactions of metastatic tumor cells and the immune cells have also been explored. Liver metastases of CRC were investigated by Wu et al. using spatial transcriptomics and multiplexed IHC [103]. These authors discovered a specific macrophage population that expresses MRC1^+^ and CCL18^+^ that resembles the resident Kupffer cells. This finding coincides with the role of the resident Kupffer cells in the primary HCC tumors [98]. These macrophages, which had anti**-**inflammatory M2-like expression features, as well as neutrophils were enriched in the liver metastases compared to the primary lesion. Spatially, these macrophages were scattered in the metastatic tumors and peritumoral boundaries. Ligand-receptor interaction analyses indicated extensive cross-talk between the metastatic tumor cells and the MRC1^+^/CCL18^+^ macrophages; an example is the “don’t-eat-me” signaling between CD47 on tumor cells and SIRPA on the macrophages.

The T cell landscape of solid tumor brain metastases was studied by Sudmeier et al. [104]. Using a spatial transcriptomics approach, they found a consistent percentage of PD-1-expressing CD8^+^ T cells among the metastatic tumor cells. They also detected variable expression of exhaustion markers and lesser T cell receptor diversity in metastatic lesions than in the primary tumor. Spatially, exhausted T cells were distributed on the periphery of the metastatic tumor rather than in the tumor core. Progenitor-like T cells were located adjacent to the inflamed peritumoral area, suggesting that differential cytokine or chemokine signaling within the tumor could serve as a differential niche for CD8^+^ T cells.

### Tumor and immune cell metabolism profiling

Tumor-specific metabolic pathways have been identified by enrichment analysis of spatially resolved transcriptomics data from ICC samples [39] and of spatially resolved metabolic analyses of esophageal squamous cell carcinomas [105]. The former study identified activation of hypoxia-related pathways and metabolism through the tricarboxylic acid cycle as well as upregulation of fatty acid metabolism components (e.g., fatty acyl CoA synthesis and fatty acid beta-oxidation of tumor cells), high proliferative capacity, and high levels of apoptosis that likely reflect higher proliferative capacity, damaged states, and energy requirements of tumor cells. The spatial metabolic platform revealed upregulation of arginine and proline metabolism, fatty acid biosynthesis, and alanine, aspartate, glutamate, pyrimidine, and histidine metabolism in tumors. Six critical metabolic enzymes within four pathways were present at higher levels in tumor compartments than in normal tissue: the pyrroline-5-carboxylate reductase PYCR2, glutaminase, uridine phosphorylase UPase1, fatty acid synthase, and ornithine decarboxylase. These enzymes catalyze proline biosynthesis, glutamine catabolism, phosphorolytic cleavage of uridine to uracil, and decarboxylation of histidine to form histamine, respectively. PYCR2 is an essential enzyme in proline biosynthesis and promotes cancer proliferation and progression [106]; however, histamine is derived from the decarboxylation of histidine, which is catalyzed by an enzyme dramatically down-regulated in cancer [107], and histamine-based therapies can lead to cancer cell apoptosis and senescence and prolong survival in tumor-bearing animals [108].

scRNA-seq and spatial transcriptomics have also been applied to a recently developed mouse model of human neuroblastoma, revealing that the spatial relationship of CD4^+^ and CCR2^+^ macrophages play a pro-tumor role via the arginine metabolic pathway [109]. Single-cell metabolic profiling also identified a tumor-specific metabolic phenotype characterized by high levels of amino acid transporter CD98 expression and showed that a tumor-associated metabolic T cell state is characterized by expression of exhaustion markers PD1 and CD39 [110] as well as downregulated levels of TCF1, which is indicative of terminal exhaustion [111].

### Intratumor cellular topologic patterns are correlated with patient prognosis

To understand the topological cellular interaction patterns in tissues, high-dimensional multi-omics data are analyzed computationally for correlations between locations of cells of particular types. The terms “cellular neighborhood” and “ecotype”, among others, have been used by independent research groups to describe the coordinated presence or absence of particular cell types [95, 98, 112]. Certain patterns of cellular coordination are linked to patient survival or prognosis in various cancers. Gouin et al. found that the CD8^+^ T cells within CDH12-enriched epithelial cellular neighborhoods in bladder tumors expressed high levels of CD49a, PD-1, or LAG3 [112]. These data may explain why the patients who have higher CDH12 levels have better response to atezolizumab treatment and longer overall survival.

The spatial topology of the TIME is also correlated with overall survival, and compartmentalization of the tumor-immune architecture is usually positively correlated with better survival. In diffuse large B cell lymphoma, Colombo et al. showed that the more structured germinal center B cell subtype was correlated with better overall survival than the dispersed subtype [113]. In CRC, tumors from the CLR subtype had more separated compartments than the tumors from patients with the DII subtype, and the presence of the compartments were statistically correlated with patient prognosis [100]. The phenomenon was also observed in the breast cancer tumors where compartmentalized score is highly correlated with overall survival [70]. In another example, Andersson et al. defined a TLS score in breast cancer specimens based on the degree of co-localization between B and T cells and found that a higher TLS score was also associated with better overall survival [96].

### Changes in compartmentalization occur during tumor progression

The development of spatial ‘omics technologies has made it possible to evaluate the spatial distribution of cell subsets, cell characteristics, and metabolism among different compartments of tumors during the course of cancer progression. For example, in HCC patients, Sheng et al. found that levels and locations of dedifferentiation, proliferation, and immune checkpoint markers evolved during the process of tumorigenesis [98]. In CRC tissue, Hartmann et al. observed metabolic polarization of immune cells toward the tumor-immune boundary with increased expression of CD98 and ASCT2 [110]. CD98 and ASCT2 are transporters with prognostic value in human cancers [114–116].

Furthermore, key genes in the progression of tumors can be identified by multi-dimensional deconstruction of spatial architecture datasets. Risom et al. obtained MIBI data on DCIS and invasive breast cancer (IBC) samples as well as normal breast tissue and extracted the top 20 most important features during the progression from DCIS to IBC [67, 117]. Hypoxia, glycolysis, stromal immune density, and desmoplasia/remodeling of the extracellular matrix were enriched in DCIS. Myoepithelial and immunoregulatory markers (e.g., PDL1, IDO1, COX2, PD1) on tumor and immune cells were enriched in IBC. Interestingly, the expression patterns of ECAD, SMA, CK5, and myoepithelial markers were similar in normal tissue and in IBC tissues, and the highest myoepithelial expression of ECAD was observed in normal breast tissue. These findings suggest that the loss of “normal” features may serve a protective function in non-progressors. Further, myoepithelial loss in the stroma surrounding cancer cells appears to induce fibroblast and immune cell activation, playing a critical role in determining clinical outcome. In summary, spatial analyses suggest that invasive progression depends on an evolving spatial distribution of multiple cell types rather than alteration in levels or distribution of a single cell subset.

## Conclusions

Studies using spatial multi-omics tools have revealed the complexity of the TIME and have shown that relative positions and interactions of cell types in the microenvironment of tumors, in addition to the cellular composition, strongly influence tumor development. A better understanding of spatial interactions is driving redefinition of tumor subtypes and shifting the focus of research to tumor-immune interaction units, the discovery of additional cell types, and the examination of the changes in the TIME compartment as cancer progresses.

Spatial ‘omics technologies are in the “boom” period of development. Scientists in this field are addressing technological obstacles that limit resolution of the platforms, multiplexing, sensitivity, and accuracy. In terms of resolution, currently commercialized spatial transcriptomic and proteomics technologies do not provide true single-cell level resolution. In several studies, parallel scRNA-seq analyses of the same specimen have been performed and used to re-assign and adjust the spatial transcriptomics data [39]. This may be a way to solve the resolution problem until higher resolution methods become widely available.

Multiplex antibody staining was a great leap forward from traditional IHC, which only allows simultaneous imaging of three or four targets. Multiplexing results in loss of sensitivity, however. For example, traditional IHC has a significantly higher detection sensitivity than cytometry-based multiplexed imaging [118]. Current multiplexed imaging protocols rely heavily on computational methods for data processing. The segmentation process, which translates the image data into cellular data, is critical and can suffer from inaccuracies. Computational segmentation requires prior selection of markers of cellular identity (e.g., nucleus and membrane border). Training data are also required to demarcate irregularly shaped or polymorphous cells (e.g., endothelial cells, fibroblasts). In addition, interpretation may be confounded by partially overlapping cells or non-specific background staining, which may affect the results of subsequent analyses and should be examined carefully before the data processing. At the time of preparation of this manuscript, new methods had recently been reported that yield three-dimensional images [119, 120]. In the future, we expect that a powerful and easy-to-handle protocol will be developed to investigate cellular heterogeneity from multilayered tumor specimens.

In the last decade, spatial ‘omics developments have led to progress toward personalized, precision medicine. Precision medicine, in which the treatment strategy is customized for each individual patient, relies heavily on detailed patient data. Thanks to technical advances, scientists are now able to analyze tumor tissue at the single-cell level with high data dimensions. The spatial multi-omics tools described in this review have revealed the heterogeneous composition of tumor and immune cells in the tumor microenvironment. These approaches have enabled comprehensive explorations of cancer and have furthered our understanding of the underlying mechanisms of tumor progression, which are critical for treatment and response. In the future, with the implementation of robust automated pipelines, clinicians and pathologists should be able to evaluate disease progression and tailor the therapeutic regimens to each patient, bringing us closer to the goal of precision medicine.

## Data Availability

The authors declare that all data supporting the findings of this study are available from the corresponding author on reasonable request.

## Abbreviations

TIME: Tumor immune microenvironment
ICBs: Immune checkpoint blockers
scRNA-seq: Single-cell RNA sequencing
CyTOF: Cytometry by time-of-flight
NK: Natural killer
IHC: Immunohistochemistry
IF: Immunofluorescence
FF: Fresh frozen
FFPE: Formalin-fixed, paraffin-embedded
NGS: Next-generation sequencing
FISH: Fluorescence in situ hybridization
HDST: High-Definition Spatial Transcriptomics
DBiT-seq: Deterministic Barcoding in Tissue sequencing
ROI: Regions of interest
DSP: Digital Spatial Profiler
FISSEQ: Fluorescent in situ sequencing
SoLiD: Sequencing by Oligonucleotide Ligation and Detection
STARmap: Spatially resolved Transcript Amplicon Readout mapping
SEDAL: Sequencing with Error-reduction by Dynamic Annealing and Ligation
MERFISH: Multiplexed error-robust FISH
smFISH: Single-molecule fluorescence in situ hybridization
MICS: MACSima Imaging Cyclic Staining
t-CycIF: Tissue-based cyclic immunofluorescence
CODEX: Co-Detection by IndEXing
Immuno-SABER: Immuno-signal amplification by exchange reaction
MOSAICA: Multi Omic Single-scan Assay with Integrated Combinatorial Analysis
FLIM: Fluorescence lifetime imaging and microscopy
IMC: Imaging mass cytometry
MIBI: Multiplex Ion Beam Imaging
MALDI: Matrix-assisted laser desorption/ionization–mass spectrometry
DESI: Desorption electrospray ionization
SIMS: Secondary ion mass spectrometry
t-MALDI: Transmission mode MALDI
AFADESI: Air flow-assisted DESI
TOF: Time-of-flight
SEAM: Spatial single nuclear metabolomics
EMT: Epithelial-mesenchymal transition
TAM: Tumor-associated macrophage
CAF: Cancer-associated fibroblast
APC: Antigen-presenting cell
MDSC: Myeloid derived suppressor cell
i-macrophage: Inhibitory macrophage
TSK: Tumor-specific keratinocyte
DCIS: Ductal carcinoma in situ
TNBC: Triple-negative breast cancer
HCC: Hepatocellular carcinoma
SCLC: Small cell lung cancer
CRC: Colorectal cancer
CLR: Crohn’s-like reaction type
DII: Diffuse inflammatory infiltration type
ICC: Intrahepatic cholangiocarcinoma
TLS: Tertiary lymphoid structure
TIL: Tumor infiltrating lymphocyte
iCAFs: Inflammatory CAFs
IBC: Invasive breast cancer
